# Beyond Necrotizing Enterocolitis Prevention: Improving Outcomes with an Exclusive Human Milk–Based Diet

**DOI:** 10.1089/bfm.2015.0134

**Published:** 2016-03-01

**Authors:** Amy B. Hair, Allison M. Peluso, Keli M. Hawthorne, Jose Perez, Denise P. Smith, Janine Y. Khan, Andrea O'Donnell, Richard J. Powers, Martin L. Lee, Steven A. Abrams

**Affiliations:** ^1^ USDA/ARS Children's Nutrition Research Center, Department of Pediatrics, Section of Neonatology, Baylor College of Medicine, Texas Children's Hospital, Houston, Texas.; ^2^ Department of Neonatology, Winnie Palmer Hospital for Women and Babies, Orlando, Florida.; ^3^ Department of Pediatrics, Northwestern University Feinberg School of Medicine, Chicago, Illinois.; ^4^ Department of Clinical Nutrition Services, Northwestern Memorial Hospital, Chicago, Illinois.; ^5^ Department of Neonatology, Good Samaritan Hospital, San Jose, California.; ^6^ Prolacta Bioscience, Industry, California.

## Abstract

***Objective:*** The aim of this study was to compare outcomes of infants pre and post initiation of a feeding protocol providing an exclusive human milk–based diet (HUM).

***Materials and Methods:*** In a multicenter retrospective cohort study, infants with a birth weight <1,250 g who received a bovine-based diet (BOV) of mother's own milk fortified with bovine fortifier and/or preterm formula were compared to infants who received a newly introduced HUM feeding protocol. Infants were excluded if they had major congenital anomalies or died in the first 12 hours of life. Data were collected 2–3 years prior to and after introduction of an exclusive HUM diet. Primary outcomes were necrotizing enterocolitis (NEC) and mortality. Secondary outcomes included late-onset sepsis, retinopathy of prematurity (ROP), and bronchopulmonary dysplasia (BPD).

***Results:*** A total of 1,587 infants were included from four centers in Texas, Illinois, Florida, and California. There were no differences in baseline demographics or growth of infants. The HUM group had significantly lower incidence of proven NEC (16.7% versus 6.9%, *p* < 0.00001), mortality (17.2% versus 13.6%, *p* = 0.04), late-onset sepsis (30.3% versus 19.0%, *p* < 0.00001), ROP (9% versus 5.2%, *p* = 0.003), and BPD (56.3% versus 47.7%, *p* = 0.0015) compared with the BOV group.

***Conclusions:*** Extremely premature infants who received an exclusive HUM diet had a significantly lower incidence of NEC and mortality. The HUM group also had a reduction in late-onset sepsis, BPD, and ROP. This multicenter study further emphasizes the many benefits of an exclusive HUM diet, and demonstrates multiple improved outcomes after implementation of such a feeding protocol.

## Introduction

Prematurity remains the leading cause of neonatal mortality in the United States. One of the most difficult aspects of the management of the premature infant is related to nutrition and gastrointestinal complications such as necrotizing enterocolitis (NEC) and intestinal perforation. The development and progression of NEC is known to cause significant morbidity and mortality. However, the exact pathophysiology of NEC has yet to be defined, thus making preventive strategies more difficult.^1–3^ Epidemiology shows that the highest-risk infants are those weighing <1,250 g, born at a gestational age of <28 weeks, and with congenital heart defects.^1^ It has been long established that breast milk is the best source of nutrition for all infants when it is safe and available. The continued gain in knowledge over the years has led the American Academy of Pediatrics (AAP) and Surgeon General to recommend in published statements the use of human milk (HM), including donor milk when needed, for premature infants.^4,5^

Over the last 10 years, the implementation of donor HM for very low birth weight (VLBW) infants has become more widespread. Hair et al. demonstrated that the use of an exclusive human milk–based diet (HUM) using mother's own milk, donor HM, and a donor HM-derived fortifier was safe and did not hinder growth of infants.^6^

There may be multiple benefits in using HM for premature infants such as lower rates of NEC, late-onset sepsis, feeding tolerance, and mortality, along with improved neurodevelopmental outcomes.^7–11^ Sullivan et al. demonstrated, in a prospective, randomized trial, that the use of breast milk and donor HM with a donor HM-derived fortifier was associated with a reduction in NEC.^12^ Given the increasing research showing reduction in NEC rates with an exclusive HUM diet, this study sought to evaluate this trend in multiple centers. The objectives were to determine whether these trends hold true across four Level 3 Neonatal Intensive Care Units at different hospitals in the United States and to evaluate other important outcomes for extremely premature infants.

## Materials and Methods

In a multicenter retrospective cohort study, infants who received a bovine-based diet (BOV) of mother's milk fortified with bovine fortifier and/or preterm formula were compared to infants who received an exclusive HUM diet feeding protocol. Infants with a birth weight (BW) <1,250 g were included. Infants were excluded if they had major congenital anomalies, died within the first 12 hours of admission, or if they were transferred in from an outside hospital after 1 week of age. Data were collected before and after the introduction of a HUM diet. For each institution below, the specific feeding protocol and time frame for data collection are detailed. In the BOV group, infants preferentially received mother's milk fortified with bovine fortifier over formula. Primary outcomes were NEC and mortality. Secondary outcomes included late-onset sepsis, retinopathy of prematurity (ROP), bronchopulmonary dysplasia (BPD), patent ductus arteriosus (PDA), intraventricular hemorrhage (IVH), ventilator days, and postmenstrual age (PMA) at discharge.

### Baylor College of Medicine

The Institutional Review Board of Baylor College of Medicine and Affiliated Hospitals in Houston, TX, approved this retrospective study. Infants were grouped based on the year of birth: 2006–2008 (BOV) and 2009–2012 (HUM). Information for coding of variables was obtained from the Vermont Oxford Network (VON).

The HUM feeding protocol was established at Texas Children's Hospital Neonatal Intensive Care Unit (NICU) in 2009 and was provided to infants <1,250 g BW. Infants received mother's own milk, donor HM, and a donor HM-derived fortifier (HUM). If mother's milk was not available then donor HM was provided after obtaining assent from the family. Enteral feeds were started with trophic feeds of 20 mL/kg/day for 3 days. Feeds were then advanced by 20 mL/kg/day as tolerated to reach a total volume goal of 140–160 mL/kg/day. Pasteurized donor HM-derived fortifier, Prolact+H^2^MF^®^ (Prolacta Bioscience, Industry, CA) was added once feeds reached 60 mL/kg/day for an additional 4 Cal/oz. At 100 mL/kg/day, fortification was increased to provide an additional 6 Cal/oz. If weight gain was <15 g/kg/day, fortification was increased to 8 Cal/oz and then 10 Cal/oz. Infants received a HUM diet until 34 weeks PMA. Prior to 2009, a feeding protocol was followed with similar advancement of the volume of feeds, but infants received mother's milk fortified with bovine fortifier and/or preterm formula (BOV). Fortification did not occur until the volume of feeds was 100 mL/kg/day in the BOV group.

### Good Samaritan San Jose

The retrospective analysis was approved by the Good Samaritan Hospital (GSH) Institutional Review Board. Initiation of the HUM diet began in August 2010, and data were collected until March 2012. The pre-protocol period of BOV diet was December 2008–July 2010 (20 months of data collection for each period).

The HUM diet was used for all VLBW infants (BW <1,000 g). Infants were fed mother's own milk or donor HM using the GSH NICU Standardized Feeding Protocol. When the protocol indicated fortification, feeds were fortified with Prolact+H^2^MF to an additional 4 Cal/oz at 100 mL/kg/day volume of feeds. Advancement to an additional 6 Cal/oz occurred when infants reached a volume of 150 mL/kg/day. If infants needed more concentrated feeds, fortification with the donor HM-derived fortifier was increased to provide an additional 8 Cal/oz. This regimen was continued until 60 days of age, and then infants were transitioned to either mother's own milk with a bovine fortifier or preterm formula if mother's own milk was not available.

### Northwestern

This retrospective study was approved by the Institutional Review Board of Northwestern University in Chicago, IL. Infants were grouped based on year of birth: January 2009–June 2012 (BOV) and July 2012–April 2014 (HUM). Information for coding of variables was obtained from the VON.

The HUM feeding protocol was established at the Prentice Women's Hospital NICU in July 2012. Infants received mother's own milk and a donor HM-derived fortifier (Prolact+H^2^MF). If mother's own milk was not available, then donor HM was provided for infants weighing ≤1,500 g, after obtaining assent from the family. Enteral feeds were started with trophic feeds of 10–20 mL/kg/day for 3 days. Feeds were advanced by 20 mL/kg/day to reach a total volume goal of 150–160 mL/kg/day. For infants ≤1,000 g Prolact+H^2^MF was added once feeds reached 100–120 mL/kg/day for an additional 4 Cal/oz. Fortification was advanced to 6 Cal/oz if weight gain was <15 g/kg/day, and further advanced to 8 Cal/oz if there was continued low weight gain. Infants were transitioned off this diet at 1,500 g or 34 weeks PMA. Prior to July 2012, a similar feeding advancement protocol was followed, but a bovine fortifier was used in place of the donor HM-derived fortifier.

### Winnie Palmer Hospital for Women and Babies

This retrospective study was approved by the Institutional Review Board of Orlando Health in Orlando, FL. Infants were grouped based on year of birth: 2008–2010 (BOV) and 2011–2013 (HUM). Information for coding of variables was obtained from the VON.

The HUM feeding protocol was initially established at the Winnie Palmer Hospital NICU in November 2009 with total adherence to the protocol in 2011. Infants received mother's own milk and/or donor milk if ≤750 g BW and ≤26 weeks gestational age. Donor HM products were provided after obtaining family consent. Enteral feeds were started with trophic feeds of 10–20 mL/kg/day for 3–5 days. Feeds were advanced by 10–30 mL/kg/day to reach a total volume goal of 150–160 mL/kg/day. HM fortifier, Prolact+H2MF was added once feeds reached 100–120 mL/kg/day for an additional 4 Cal/oz. If weight gain was determined to be suboptimal based on growth curve velocity by the attending physician, an additional 2–4 Cal/oz of fortification was added to the feeds for a total of 6–8 kcal/oz. Infants were transitioned off the diet at approximately 32 weeks PMA. Prior to 2009, babies received mother's own milk with bovine fortifier and/or preterm formula. Bovine fortifier was introduced at approximately 120–150 mL/kg/day.

### Definitions

Small for gestational age (SGA) was defined as a weight of <10th percentile on the Fenton Growth Curve.^13^ All NEC and spontaneous intestinal perforation cases in the VON were reviewed individually at the time of data collection for this retrospective study. A medical NEC diagnosis was designated as Bell's stage IIA or greater^14^ with the presence of pneumatosis on abdominal radiograph as determined by a pediatric radiologist. The determination of NEC was made at each of the study sites by agreement within those sites between the neonatologist and the radiologist. Both radiographic and clinical correlates of Bell's stage IIA or greater were used at all sites to define NEC cases. Surgical NEC was defined as requiring surgical intervention within the acute phase of illness. Late-onset sepsis was defined by the VON as any blood culture and/or cerebrospinal fluid positive microbial infection after 3 days of life. Severe ROP (threshold ROP) was defined as stage 3–5. Grade 3–4 IVH was classified by a pediatric radiologist per classifications by Papile et al.^15^ As per the VON definition, BPD was defined as need for oxygen at 36 weeks PMA.

### Analysis

All analyses were based on the intent-to-treat principle. Descriptive statistics were used to summarize quantitative variables and reported as mean ± standard deviation or with median for skewed data. Initially, analyses were performed on a univariate basis. Categorical data were compared between the study groups using the chi-square test for univariate analysis. Quantitative data were compared using the Wilcoxon rank sum test because of the right skew of some of the data. Comparisons were considered statistically significant at *p* < 0.05. An additional multivariate adjustment model was considered for many of the binary endpoints (including NEC, surgical NEC, late-onset sepsis, IVH, ROP, PDA, and BPD) with the use of antenatal steroids and/or study site as adjustment covariates. Study site was adjusted for in a multiple logistic regression model with group (BOV versus HUM) as the main effect. Indicator variables were included in the multivariate adjustment model for study sites. A similar adjustment model based on a log or square root-transformed linear regression model was employed for ventilation days, length of stay, and PMA at discharge.

## Results

The four centers had 1,587 infants eligible for inclusion in the study. There were 768 infants in the BOV group and 819 infants in the HUM group. The number of infants from each study site were as follows: Baylor College of Medicine 978, Good Samaritan San Jose 91, Northwestern 317, and Winnie Palmer Hospital for Women and Babies 201. The characteristics of the two groups were similar (Table 1). However, there was statistically significant difference in BW and birth head circumference. The BOV group had a 5% lower percentage of mothers receiving antenatal steroids. There were no differences in APGAR scores between the two groups (Table 1).

**Table 1.** Infant Characteristics

	*BOV (*n* = 768)*	*HUM (*n* = 819)*	p*-Value*
Male (%)	49.5	50.2	0.78
Gestational age (weeks)	26.4 ± 2.3	26.5 ± 2.5	0.24
Birth weight (g)	823 ± 205	844 ± 210	0.047
Birth head circumference (cm)	23.6 ± 2.2	23.8 ± 2.2	0.045
SGA at birth (%)	17.0	17.8	0.67
Major birth defects (%)	6.0	5.7	0.78
APGAR 5 minutes <4 (%)	8.1	8.5	0.77
Antenatal steroids (%)	75.2	80.5	0.01
African American race (%)	33.3	33.5	0.93

BOV, bovine-based diet; HUM, exclusive human milk–based diet; SGA, small for gestational age (weight <10th percentile on Fenton Growth Curve^13^).

**FIG. 1.**
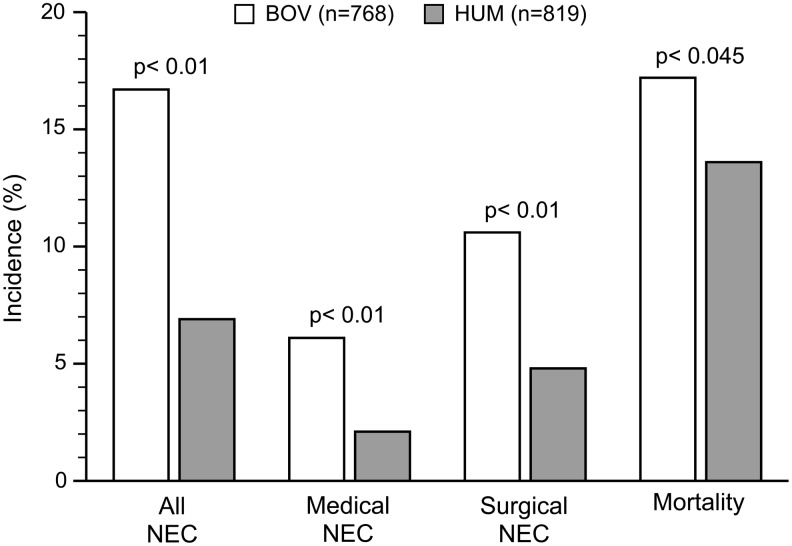
Necrotizing enterocolitis and mortality rates. BOV, bovine-based diet; HUM, exclusive human milk-based diet; NEC, necrotizing enterocolitis.

**FIG. 2.**
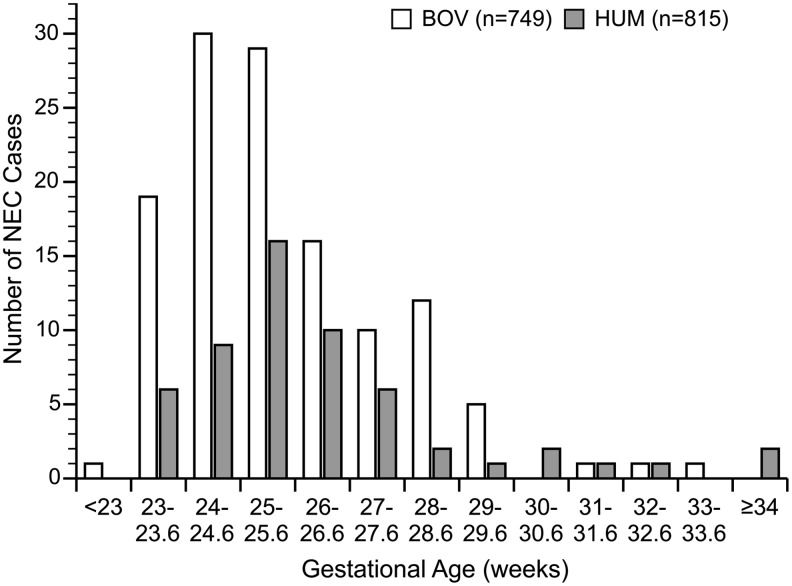
Distribution of cases of necrotizing enterocolitis by gestational age.

The primary outcomes, NEC and mortality, were statistically significant (Table 2). The BOV group mortality rate was 17.2% (132/768), and the HUM group rate was 13.6% (111/819; *p* = 0.04). There was a reduction in NEC and/or death (*p* = 0.0002) that persisted when adjusted by study site. There was a significant reduction in NEC cases in the HUM group (Fig. 1). The number needed to treat to prevent one case of NEC is 10. With the degree of reduction in NEC cases, a subgroup analysis of NEC patients was completed (Table 3). There was a significant reduction in medical and surgical NEC (*p* < 0.00005 and *p* < 0.00002). The distribution of the number of NEC cases by gestational age is shown in Figure 2.

**Table 2.** Primary Outcomes

	*BOV (*n* = 768)*	*HUM (*n* = 819)*	p*-Value*
NEC (%)	16.7	6.9	<0.00001
Mortality (%)	17.2	13.6	0.04
NEC and/or mortality (%)	28.0	18.2	<0.00001
Weight gain (g/day)	19.5 ± 8.4	20.3 ± 8.6	0.08
Head circumference growth (cm/week)	0.71 ± 0.33	0.69 ± 0.26	0.22
SGA at discharge (%)	48.6	50.3	0.51

NEC, necrotizing enterocolitis.

**Table 3.** Outcomes Among Patients with NEC

	*BOV (*n* = 125)*	*HUM (*n* = 56)*	p*-Value*
Medical NEC (%)	6.1	2.1	0.00005
Surgical NEC (%)	10.6	4.8	0.00002
Mortality (%)	33.9	32.1	0.82
Length of stay (days)	112 ± 69	105 ± 67	0.53
Late-onset infection (%)	52.0	47.3	0.56
SGA at discharge (%)	56.5	64.6	0.36

The secondary outcomes considered include growth parameters as well as variables associated with neonatal morbidity (Table 4). There was no statistical increase in weight gain, head circumference growth, or being SGA at the time of discharge (Table 2). There was no significant decrease in severe IVH, length of stay, or PMA at discharge. However, there was a significant reduction in the incidence of late-onset infection, ROP, presence of PDA, and BPD (Table 4). Using the Wilcoxon rank sum test, there was a reduction in ventilator days from a mean of 32 days with a median of 17 days to a mean of 29 days with a median of 9 days (*p* = 0.003).

**Table 4.** Secondary Outcomes

	*BOV (*n* = 768)*	*HUM (*n* = 819)*	p*-Value*
Late-onset infection (%)	30.3	19.0	<0.00001
IVH: Grade 3 or 4 (%)	16.8	14.5	0.22
Ventilator (days)	32.2 ± 44.9	29.3 ± 44.2	0.003
Length of stay (days)	94.7 ± 62.1	92.4 ± 54.4	0.44
Postmenstrual age at discharge (weeks)	40.1 ± 8.8	39.4 ± 7.5	0.10
Retinopathy of prematurity (%)	9.0	5.2	0.003
Patent ductus arteriosus (%)	64.7	55.1	0.0001
Bronchopulmonary dysplasia (%)	56.3	47.7	0.0015

IVH, intraventricular hemorrhage.

Both logistic (categorical outcomes) and multiple linear regression (quantitative outcomes) models were used to adjust for the use of antenatal steroids. The model consisted of the particular primary or secondary outcome as the dependent variable, and study group (HUM or BOV) along with antenatal steroid use as the independent variables. Significance was lost only for mortality (*p* = 0.27). All other outcomes remained statistically significant. These models were also used to adjust for study site, and all variables remained significant.

## Discussion

It was found that the use of an exclusive HUM diet in extremely premature infants (<1,250 g BW) decreased the incidence of both medical and surgical NEC, further supporting the results of Sullivan et al. in regards to reduction of NEC.^12^ This study also shows an association with a reduction in mortality and late-onset infections. An association was demonstrated with decreased ROP, BPD, PDA, and ventilator days that has not been previously reported. In addition to the reduction in NEC, the additional reduction in morbidity may decrease the cost of a NICU hospitalization. Recent analysis found a significant cost reduction when using a HUM feeding protocol compared with using a bovine fortifier.^16^

To the authors' knowledge, this is the first study to compare rates of NEC after a feeding protocol implementation at multiple institutions with multiple years of follow-up while using an exclusive HUM diet. Additional strengths of this study include a large study population to evaluate differences in outcomes between the two groups. The multiple centers involved increases the generalizability of the study.

In addition, while many clinical studies have extensive exclusion criteria, including inability to obtain parental consent, this study only excluded infants with major congenital anomalies, infants who died within 48 hours of admission, or infants transferred to another hospital after 1 week of age. Therefore, all infants <1,250 g BW were included in this naturalistic study, including infants <23 weeks of age, <500 g BW, and growth-restricted infants. Due to the inclusion of high-risk infants, who are not often included in feeding or NEC studies, it is speculated that this may have contributed to the higher rates of NEC, sepsis, and mortality. However, a significant reduction was shown in all outcomes after the introduction of an exclusive HUM diet. Although three of the four centers fed infants <1,000 g BW a HUM feeding protocol, 62% of the infants in this study were from Baylor College of Medicine (Texas Children's Hospital), which fed infants <1,250 g BW a HUM feeding protocol. The effect of a HUM diet provides benefits across all weight categories consistent with the previous randomized clinical trials evaluating a HUM diet.^9,12^

Some limitations to this study are acknowledged. A small difference was identified in BW and birth head circumference between study groups that is not believed to be clinically significant. The nature of a retrospective study lends to several biases such as misclassification biases, particularly in relation to the diagnosis of NEC, although the classification was determined by two researchers separately. It is possible that over the period this study occurred, there were changes in practice aside from type of milk and fortifier used that could confound these notable changes. It is acknowledged that mother's milk and pasteurized donor HM likely increased during the study period and potentially contributed to the improved outcomes. However, the study showed a 60% decrease in the incidence of NEC compared with only a 20% decrease in NEC in participating centers in the VON over the same time period. It is also acknowledged that at the time of the feeding protocol changes, there was increased awareness of central line infection initiatives to decrease central line associated bloodstream infections, which may have contributed to a reduction in late-onset infection. However, an all-HUM diet has been shown to be associated with decreased parenteral nutrition days, which serves as a surrogate for central line days.^6,9^ In addition, with a decrease in the incidence of NEC, it was possible to reduce the central line days.

## Conclusion

The use of an exclusive HUM diet is associated with significant benefits for extremely premature infants <1,250 g BW. The benefits include decreased NEC rates, mortality, late-onset sepsis, PDA, BPD, ventilator days, and ROP. Importantly, while evaluating the benefits of using an exclusive HUM-based protocol, it appears that there were no feeding-related adverse outcomes. This study demonstrates that an exclusive HUM diet provides important benefits beyond NEC.

## References

[1] Sharma R, Hudak ML A clinical perspective of necrotizing enterocolitis: past, present, and future. Clin Perinatol 2013;40:27–51 23415262 10.1016/j.clp.2012.12.012 PMC3575605

[2] Huda S, Chaudhery S, Ibrahim H, et al. Neonatal necrotizing enterocolitis: clinical challenges, pathophysiology and management. Pathophysiology 2014;21:3–12 24525171 10.1016/j.pathophys.2013.11.009

[3] Yee WH, Soraisham AS, Shah VS, et al. Incidence and timing of presentation of necrotizing enterocolitis in preterm infants. Pediatrics 2012;129:e298–304 22271701 10.1542/peds.2011-2022

[4] American Academy of Pediatrics Section on Breastfeeding. Breastfeeding and the use of human milk. Pediatrics 2012;129:e827–84122371471

[5] McGuire S U.S. Dept. of Health and Human Services. The Surgeon General's Call to Action to Support Breastfeeding. U.S. Dept. of Health and Human Services, Office of the Surgeon General. 2011. Adv Nutr 2011;2:523–524 22332095 10.3945/an.111.000968 PMC3226390

[6] Hair AB, Hawthorne KM, Chetta KE, et al. Human milk feeding supports adequate growth in infants ≤1250 grams birth weight. BMC Res Notes 2013;6:459 24220185 10.1186/1756-0500-6-459 PMC3879715

[7] Abrams SA, Schanler RJ, Lee ML, et al. Greater mortality and morbidity in extremely preterm infants fed a diet containing cow milk protein products. Breastfeed Med 2014;9:281–285 24867268 10.1089/bfm.2014.0024 PMC4074755

[8] Arslanoglu S, Ziegler EE, Moro GE Donor human milk in preterm infant feeding: evidence and recommendations. J Perinat Med 2010;38:347–351 20443660 10.1515/jpm.2010.064

[9] Cristofalo EA, Schanler RJ, Blanco CL, et al. Randomized trial of exclusive human milk versus preterm formula diets in extremely premature infants. J Pediatr 2013;163:1592–1595 23968744 10.1016/j.jpeds.2013.07.011

[10] Ghandehari H, Lee ML, Rechtman DJ An exclusive human milk-based diet in extremely premature infants reduces the probability of remaining on total parenteral nutrition: a reanalysis of the data. BMC Res Notes 2012;5:188 22534258 10.1186/1756-0500-5-188 PMC3527141

[11] Herrmann K, Carroll K An exclusively human milk diet reduces necrotizing enterocolitis. Breastfeed Med 2014;9:184–190 24588561 10.1089/bfm.2013.0121 PMC4025624

[12] Sullivan S, Schanler RJ, Kim JH, et al. An exclusively human milk-based diet is associated with a lower rate of necrotizing enterocolitis than a diet of human milk and bovine milk-based products. J Pediatr 2010;156:562–567 e1. 20036378 10.1016/j.jpeds.2009.10.040

[13] Fenton T A new growth chart for preterm babies: Babson and Benda's chart updated with recent data and a new format. BMC Pediatr 2003;3(13) 10.1186/1471-2431-3-13 PMC324406 14678563

[14] Bell MJ, Ternberg JL, Feigin RD, Keating JP, et al. Neonatal necrotizing enterocolitis. Therapeutic decisions based upon clinical staging. Ann Surg 1978;187:1–7 413500 10.1097/00000658-197801000-00001 PMC1396409

[15] Papile LA, Burstein J, Burstein R, et al. Incidence and evolution of subependymal and intraventricular hemorrhage: a study of infants with birth weights less than 1,500 gm. J Pediatr 1978;92:529–534 305471 10.1016/s0022-3476(78)80282-0

[16] Ganapathy V, Hay JW, Kim JH Costs of necrotizing enterocolitis and cost-effectiveness of exclusively human milk-based products in feeding extremely premature infants. Breastfeed Med 2012;7:29–37 21718117 10.1089/bfm.2011.0002

